# Correlation of anti-Müllerian hormone levels with metabolic syndrome events in polycystic ovary syndrome: A cross-sectional study

**DOI:** 10.18502/ijrm.v18i3.6716

**Published:** 2020-03-29

**Authors:** Budi Wiweko, Lieke Koes Handayani, Achmad Kemal Harzif, Gita Pratama, Raden Muharam, Andon Hestiantoro, Kanadi Sumapraja

**Affiliations:** ^1^Division of Reproductive Endocrinology and Infertility Department of Obstetrics and Gynecology, Faculty of Medicine Universitas Indonesia, Jakarta, 10430, Indonesia.; ^2^Yasmin IVF Clinic, Dr. Cipto Mangunkusumo General Hospital, Jakarta, 10430, Indonesia.; ^3^Human Reproductive, Infertility and Family Planning Research Center, Indonesia Medical Education and Research Institute (IMERI), Faculty of Medicine, Universitas Indonesia, Jakarta, 10430, Indonesia.

**Keywords:** Anti-Müllerian hormone, Metabolic syndrome, Polycystic ovary syndrome.

## Abstract

**Background:**

Various endocrine disorders have been reported in women of reproductive age, 10% of which is affected by polycystic ovary syndrome (PCOS).

**Objective:**

This study aimed to evaluate the correlation of anti-Müllerian hormone (AMH) levels with the metabolic syndrome in patients with PCOS.

**Materials and Methods:**

This cross-sectional study employed a consecutive sampling method using medical records from January 2013 to December 2017 at Dr. Cipto Mangunkusumo General Hospital polyclinic and Yasmin in vitro fertilization Clinic (Kencana), Jakarta, Indonesia. The primary outcome of the study was the AMH levels as independent variable correlated with metabolic syndrome. The secondary outcome was also the AMH levels correlated with each PCOS phenotype. The tertiary outcome was each PCOS phenotype as independent variable correlated with metabolic syndrome.

**Results:**

Women with phenotype 1 of PCOS had a median AMH level of 13.92 (range: 3.88-34.06) ng/ml. 21% patients had metabolic syndrome, with a median AMH level 7.65 (3.77-20.20) ng/ml, higher than the women without metabolic syndrome (p = 0.38). The most frequent phenotype in women with PCOS was phenotype 4, oligo- or anovulation and polycystic ovary morphology (OA/PCOM) in 41.3%. The most frequent phenotype in women with metabolic syndrome was phenotype 1, OA + PCOM + hyperandrogenism in 56.5%.

**Conclusion:**

All PCOS phenotypes exhibited significant correlations with the AMH level. Phenotype 1 (OA + PCOM + hyperandrogenism) was associated with the highest AMH level and was significantly associated with metabolic syndrome.

## 1. Introduction

Various endocrine disorders have been reported in women of reproductive age; approximately 10% of them have been reported to be affected by polycystic ovary syndrome (PCOS) (1, 2). According to the 2003 Rotterdam consensus, PCOS is diagnosed by the presence of 2 of the following 3 criteria: oligo- and/or anovulation (OA); hyperandrogenism (HA), which is defined as hirsutism (Ferriman-Gallwey score (FG index) > 5); and the identification of polycystic ovary morphology (PCOM) on an ultrasound examination, defined as a minimum of 12 follicles with diameters of 2-9 mm per ovary and/or an increased ovarian volume (minimum: 10 mm${}^{3}$). Based on these criteria, patients with PCOS can be subdivided into four phenotypic groups: phenotype 1, OA + HA + PCOM; phenotype-2, OA + HA; phenotype-3, PCOM + HA; and phenotype-4, OA + PCOM (3-5).

“The Modified National Cholesterol Education Program Adult Treatment Panel (NCEP ATP III) defined metabolic syndrome as the presence of three of the following five criteria: waist circumference of > 80 cm, blood pressure of > 130/85 mmHg, fasting triglyceride (TG) level of > 150 mg/dl, fasting high-density lipoprotein (HDL) cholesterol level of < 50 mg/dl, and fasting blood sugar of > 100 mg/dl” (6, 7).

Follicle development is regulated by anti-Müllerian hormone (AMH), also known as transforming growth factor (TGF)-β. This hormone inhibits primordial follicle growth by decreasing the sensitivity of the follicles to follicle-stimulating hormone (FSH), resulting in the pooling of small antral follicles (8). Because AMH regulates follicle growth, it is considered to be a marker of ovarian reserve. A higher level of plasma AMH indicates the severity of PCOS (9).

Our study aimed to evaluate the correlation of anti-Müllerian hormone (AMH) levels with the metabolic syndrome in patients with PCOS.

## 2. Materials and Methods

In this cross-sectional study, medical records of 109 PCOs women with available AMH data referred to Dr. Cipto Mangunkusumo General Hospital (RSCM) polyclinic and Yasmin in vitro fertilization Clinic (Kencana), Jakarta, Indonesia from January 2013 to December 2017 were investigated. Data collection was performed using consecutive sampling method. The inclusion criteria were women in reproductive age (15-45 years) and the presence of the Rotterdam criteria for PCOS and NCEP ATP III criteria for metabolic syndrome. Participants were classified by PCOS phenotypes into 4 groups. Information on the levels of AMH, BMI, waist circumference, blood pressure, trygliceride, HDL, Ferrimen-Galwey (FG)-index, hypertension, diabetes mellitus (DM), and metabolic syndrome were extracted from participants' record and recorded. The primary outcome of the study was the AMH levels as independent variable correlated with metabolic syndrome. The secondary outcome was also the AMH levels correlated with each PCOS phenotype. The tertiary outcome was each PCOS phenotype as independent variable correlated with metabolic syndrome.

### Ethical consideration

This study was approved by the Ethics Committee of the Faculty of Medicine (reference number: 30/UN2.F1/ETIK/2015). Informed consent was obtained from every participant prior to study enrollment.

### Statistical analysis

Statistical analyses were done using SPSS, version 22 (Statistical Package for the Social Sciences, version 22.0, SPSS Inc, Chicago, Illinois, USA). Abnormally distributed data are presented as medians (ranges) and were analyzed statistically using the Mann-Whitney test, independent *t* test, Chi-square test, and Fisher's exact test. Additionally, 95% confidence intervals (CI) were calculated for the study data and p < 0.05 to be statistically significant.

## 3. Results

The study samples included 109 PCOs women with available AMH data. According to our results, phenotype-1 was the most frequent PCOS phenotype in women with metabolic syndrome (56.5%), whereas phenotype-4 was most frequent in women without metabolic syndrome (43.1%) (Table I). As shown in Table II, the PCOS phenotype-1 group had the highest frequency of metabolic syndrome (36.1%), and the highest AMH level, 12.99 (3.88-34.06) ng/ml. The phenotype-3 group had the highest FG index (7), while the phenotype-4 group had the highest frequencies of DM. Further, the phenotype-1 group had the highest frequencies of hypertension. An independent *t* test revealed significant differences in the AMH levels between the different PCOS phenotypes. Phenotype-1 was the most frequent and the most associated with the highest AMH level (Table III).

A Mann-Whitney test was then applied to test the association between the AMH level and metabolic syndrome status. Notably, 21% of participants had metabolic syndrome, with a median AMH level of 7.65 (3.77-20.20) ng/ml compared to 79% of participants without metabolic syndrome who had a median AMH level of 7.05 (3.11-34.06) ng/ml. Although, these differences was not significant (p > 0.05). A linear regression analysis revealed significant correlations of age and HDL with the AMH level as a predictor of metabolic syndrome with p = 0.004 and p = 0.034, respectively. In this analysis, each one-year increase in age would decrease the AMH level by 0.58-fold. Each 1 mg/dL increase in the HDL level would increase the AMH level by 0.19 fold. As shown in table IV, a significant correlation was observed between PCOS phenotype-1 and metabolic syndrome (p = 0.007. The highest frequency of metabolic syndrome, 36.1%, was observed in the group of patients with phenotype-1.

**Table 1:** Baseline characteristics of study participants


**Characteristic**		**With metabolic syndrome median (range)**	**Without metabolic syndrome median (range)**
**Age, years**		30 (19-41)	32 (18-44)
**BMI, kg/m${}^{2}$**		28.89 (21.90-38.86)	26.34 (17.40-44.33)
**Waist circumference, cm**		86.0 (79.0-103.0)	82.5 (57.0-114.0)
**Systole, mmHg**		127 (102-150)	111 (93-135)
**Diastole, mmHg**		83 (70-100)	73 (60-94)
**Fasting blood glucose, mg/dL**		96 (68-271)	86 (68-132)
**Triglyceride, mg/dL**		169 (83-473)	95 (41-156)
**HDL, mg/dL**		40 (21-51)	48 (26-64)
**FG Index**			
	**< 5**	4 (36.4%)	7 (63.6%)
	**≥ 5**	14 (22.6%)	48 (77.4%)
**PCOs phenotype**			
	**1: OA + HA + PCOM**	13 (56.5%)	23 (26.7%)
	**2: OA + HA**	1 (4.4%)	11 (12.8%)
	**3: PCOM + HA**	1 (4.4%)	15 (17.4%)
	**4: OA + PCOM**	8 (34.7%)	37 (43.1%)
BMI: Body mass index; HDL: High-density lipoprotein; FG: Ferriman-Gallwey; OA: Oligo- or anovulation; HA: Hyperandrogenism; PCOM: Polycystic ovary morphology			

**Table 2:** Baseline characteristics by PCOS phenotype and metabolic syndrome status


**Characteristic**	**PCOS** **phenotype**			
	**Phenotype-1**	**Phenotype-2**	**Phenotype-3**	**Phenotype-4**
**Age, years**	30 (18-44)	32 (27-37)	34 (26-40)	32 (20-41)
**BMI, kg/m${}^{2}$**	27.60 (17.40-38.20)	26.23 (20-36)	22.85 (19.50-40.52)	27.40 (20.20-44.33)
**Waist circumference, cm**	88 (62-107)	82 (58-105)	66.5 (57-100)	84 (61-114)
**Systolic blood pressure, mmHg**	120 (93-142)	116.5 (110-140)	110 (100-135)	111 (94-150)
**Diastolic blood pressure, mmHg**	78 (64-94)	80 (70-90)	80 (69-90)	74 (60-100)
**Fasting blood glucose, mg/dL**	88 (68-126)	87.5 (68-110)	86 (82-132)	86 (72-271)
**Triglyceride, mg/dL**	109 (45-473)	117 (72-169)	156 (90-174)	95.5 (41-422)
**HDL, mg/DL**	41 (21-53)	48 (42-62)	44 (37-49)	51 (32-64)
**Metabolic syndrome**	13 (36.1%)	1 (8.3 %)	1 (6.2%)	8 (17.8%)
**FG index**	6 (1-14)	6 (4-8)	7 (1-14)	2.5 (0-4)
**Hypertension**	5 (13.9%)	1 (8.3 %)	1 (6.2%)	5 (11.1%)
**DM**	5 (14.3%)	1 (8.3%)	2 (12,5%)	8 (20%)
**AMH, ng/ml**	12.99 (3.88-34.06)	4.05 (3.11-15)	4.98 (4.05-8.60)	6.49 (3.7-18.9)
*BMI: Body mass index; HDL: High-density lipoprotein; AMH: Anti-Müllerian hormone; FG: Ferriman-Gallwey; DM: Diabetes mellitus; PCOS: Polycystic ovary syndrome				

**Table 3:** Correlations of AMH levels with polycystic ovary syndrome phenotypes


	**n (%)**	**AMH [median (min-max)]**	**P-value**
**Phenotype 1: OA + HA + PCOM**	36 (33)	13.92 (3.88-34.06)	<0.001
**Phenotype 2: OA + HA**	12 (11)	4.05 (3.11-15.0)	0.004
**Phenotype 3: PCOM + HA**	16 (14.7)	4.70 (4.05-8.60)	0.005
**Phenotype 4: OA + PCOM**	45 (41.3)	6.49 (3.70-17.59)	0.023
*AMH: Anti-Müllerianhormone; OA: Oligo-or anovulation; HA: Hyperandrogenism; PCOM: Polycystic ovary morphology **Independent *t* test			

**Table 4:** Correlations of PCOS phenotypes with metabolic syndrome


**Variables**	**Metabolic syndromes**				**P-value**	**OR**	**95% CI**	
	**Yes**		**No**			**Min**	**Max**
	**n**	**%**	**n**	**%**		
**Phenotype 1: PCOM + Anovulation + Hyperandrogenism **	13	36.1	23	63.9	0.007*	3.56	0.10	9.23
**Phenotype 2: Anovulation + Hyperandrogenism**	1	8.3	11	91.7	0.454${}^{\#}$	0.31	0.04	2.54
**Phenotype 3: PCOM + Hyperandrogenism**	1	6.2	15	93.8	0.184${}^{\#}$	0.22	0.03	1.72
**Phenotype 4: PCOM + Anovulation**	8	17.8	37	82.2	0.476*	0.71	0.27	1.84
**Total**	23	21.10	86	78.90		
*Chi-square test; ${}^{\#}$Fisher's exact test								

## 4. Discussion

In this study, 23% of women with PCOS had metabolic syndrome, compared to reported rates of 8.2%, 14.5%, 30.6%, and 46% from studies performed in Italy, Korea, South India, and the USA, respectively (4, 10-12). These inter-study differences may be attributable to differences in lifestyles, diets, and the criteria used to define metabolic syndrome. Moreover, we observed a median AMH level of 7.65 ng/ml in women with metabolic syndrome, which did not differ significantly from the level of 7.05 ng/ml in those without metabolic syndrome (p = 0.387). This observation is consistent with the findings from previous studies by Wiweko and colleagues and Lin and colleagues which non-significantly higher levels of AMH were observed in women with metabolic syndrome. Interestingly, in women, PCOS has been associated with dyslipidemia, as the high level of androgens increases the risk of atherosclerosis. AMH levels have also been observed to correlate with markers of metabolic syndrome, such as the HDL and triglyceride levels (11, 12).

In our study, more than half of the women with PCOS phenotype-1 were found to have metabolic syndrome. Accordingly, this was considered the group most likely to present with metabolic syndrome. Our finding was similar to that of a study conducted in Poland by Gluszak and colleagues, who reported phenotype 1 as the most frequent (60.1%). Numerous other studies have also reported that phenotype-1 was the most prevalent and was associated with insulin resistance (8, 9, 13-14).

The highest median AMH levels were also observed in women with PCOS phenotype-1 (13.92 ng/ml), and this level was significantly higher than the levels observed in other phenotype groups (p < 0.01). Normally, the preantral follicle and small antral follicle are the greatest producers of AMH. However, once the follicle diameter exceeds 8 mm, AMH production is reduced and the follicular sensitivity to FSH increases. However, AMH levels 75 times greater than normal were observed in the granulosa cell masses of patients with PCOS, and this phenomenon may be a potential contributor to anovulation. Still, the underlying cause of increased AMH production remains unknown, although several other signals produced by granulosa cells and oocytes may modulate the ovarian environment and affect oocyte maturation (8).

In our study, we found that metabolic syndrome is most common among women with PCOS phenotype-1, followed by phenotypes-4, 2, and 3. These results differ from those of previous studies that observed the occurrence of metabolic syndrome in association with many hyperandrogenic conditions (15, 16). Similarly, normoandrogenic PCOS was associated with relatively lower risks of metabolic syndrome, cardiovascular risk factors, and insulin sensitivity when compared with hyperandrogenic PCOS (7). Furthermore, our phenotypic analyses revealed that women with metabolic syndrome mostly presented with PCOS phenotypes 1 (36%) and 4 (17.8%), and both phenotypes were associated with relatively larger median waist circumferences (94 and 85 cm, respectively) when compared to those of patients with phenotypes-2 and 3. Intra-abdominal fat is the greatest contributor to the turnover of free fatty acids derived from brown adipose tissue and subsequent distribution to other body organs. Intra-abdominal fat also secretes adipocytokines, such as leptin, adiponectin, resistin, and interleukins (IL-1 and IL-6), which act as energy regulators and inflammatory markers and are thus correlated strongly with metabolic syndrome (17).

We further identified a statistically significant association between increased AMH levels and metabolic syndrome (p = 0.03). The observed inverse relationship between age and AMH levels reflects how the ability of a woman to produce oocytes of good quality and quantity decreases with age. Our multiple linear regression further identified statistically significant relationships of age with the AMH and HDL concentrations. The observed linear relationship between AMH and HDL is consistent with the findings of a study by Yarde and colleagues, who reported that increases in HDL-C up to 1.6 mmol/L were associated with increases in AMH to 3.0 ng/ml (18).

## 5. Conclusion

In summary, AMH can be used as a marker for metabolic syndrome, especially in phenotype 1 as it was associated with insulin resistance. Further research with a better research design is needed to enhance our results.

##  Conflict of Interest

The authors declare that they have no competing interests.
